# LncRNA KCNQ10T1 shuttled by bone marrow mesenchymal stem cell-derived exosome inhibits sepsis via regulation of miR-154-3p/RNF19A axis

**DOI:** 10.1007/s00441-023-03784-4

**Published:** 2023-06-16

**Authors:** Haojie Yuan, Junbo Yu, Chun Liu, Heyan Zhao, Jianhua Xue, Jiajia Liu, Yang Yang

**Affiliations:** 1 Department of Trauma Center, Affiliated Hospital of Nantong University, Chongchuan District, Jiangsu Province No. 20 Xisi Road, 226001 Nantong, China; 2 Department of Emergency Medicine, Affiliated Hospital of Nantong University, Jiangsu Province, Nantong, 226001 China; 3 Department of Human Anatomy, Institute of Neurobiology, Building of Qixiu Campus, Medical School of Nantong University, Jiangsu Province, No.19 Qixiu Road, Nantong, 226001 No.3 China

**Keywords:** Sepsis, Long non-coding RNA, KCNQ1 opposite strand/antisense transcript 1, MiR-154-3p, RNF19A

## Abstract

This study aims to discuss the role of exosomes KCNQ10T1 derived from bone marrow mesenchymal stem cells (BMMSCs) in sepsis and to further investigate its potential molecular mechanisms. Exosomes extracted from BMMSCs are identified by transmission electron microscopy (TEM), nanoparticle tracking analysis (NTA), and western blot. Fluorescence labeling is applied to detect the internalization of exosomes in receptors. The proliferation ability, migration ability, and invasion ability of HUVECs are determined by CCK-8, EdU, wound healing, and Transwell. The levels of inflammatory cytokines in sepsis cells are quantitatively detected by ELISA. Kaplan–Meier survival curve is used to describe the overall survival. RT-qPCR is used to detect mRNA expression of related genes. Bioinformatics analysis is performed to search the downstream target of KCNQ1OT1 and miR-154-3p and the interaction is verified by luciferase reporter assay. Exosomes derived from BMMSCs alleviated the toxicity in sepsis cell models and animal models. In mice with septic cell models, exosomal KCNQ10T1 was down-regulated and associated with lower survival. Overexpression of KCNQ10T1 inhibited the proliferation and metastasis of LPS-induced HUVECs. Further research illustrated that miR-154-3p was the downstream target gene of KCNQ1OT1 and RNF19A was the downstream target gene of miR-154-3p. Importantly, functional research findings indicated that KCNQ1OT1 regulated sepsis progression by targeting miR-154-3p/RNF19A axis. Our study demonstrates that the exosomal KCNQ1OT1 suppresses sepsis via mediating miR-154-3p/RNF19A, which provides a latent target for sepsis treatment.

## Introduction

Sepsis is a syndrome in which the human body causes an excessive response to various infectious diseases. Patients with sepsis show a series of symptoms such as high fever, nausea, vomiting, and even shock (Alkharfy et al. 2018). If sepsis cannot be treated in a timely and effective manner, it can lead to multiple organ dysfunction and severe diseases in the most severe cases, and it is also accompanied by severe immune dysfunction and catabolism (Seymour et al. 2014). In China, the incidence of sepsis is on the rise, and sepsis-related deaths are also quite high (Yong et al. 2020). Currently, the therapeutic strategies for sepsis are mainly antimicrobial therapy and fluid resuscitation therapy. However, most clinical trials using antimicrobial drugs are failed in improving survival in patients with sepsis, and fluid overload can be detrimental to the heart (Boyd et al. 2011; Essandoh et al. 2015). Therefore, it was urgent to explore new treatments for sepsis. Mesenchymal stem cells (MSCs) and their derived exosomes have shown good potential in controlling CLP treatment leading to sepsis (Pei et al. 2021﻿﻿).

Bone marrow mesenchymal stem cells (BMMSC) have self-renewal and multidirectional differentiation potential. Therefore, the therapeutic potential of BMMSC for a variety of diseases has been deeply investigated (Chen et al. 2020; Shen et al. 2013). In addition, BMMSC has also been shown to interact with immune cells and participate in immune regulation (Le Blanc et al. 2003). Based on this immunomodulatory potential, it has been proposed that the therapeutic potential of BMMSCs is usually based on paracrine rather than cell-dependent approaches (Borger et al. 2017). In recent years, it has become increasingly obvious that the therapeutic active ingredient of BMMSC is not only soluble factors but vesicle structures-exosomes (Phinney et al. 2015). Exosomes are endogenous structures that are secreted into the extracellular space. In the extracellular environment, these functional biomolecules are stably protected from external degrading enzymes through the lipid bilayer of the exosome membrane to mediate cell-to-cell micro communication, immune regulation, and tissue regeneration (Kim et al. 2016). In addition, studies have shown that the exosome-derived lncRNA neat1 promotes iron death by regulating the miR-9-5p/TFRC and got1 axis, thereby exacerbating sepsis-associated encephalopathy (Wei et al. 2022).

Long non-coding RNAs (lncRNAs) are transcripts containing over 200 nt unable to encode proteins (Dey et al. 2014). Studies have shown that abnormal expression of lncRNA is associated with the occurrence of many diseases, including sepsis (Yang et al. 2018). Besides, increasing evidence has shown that lncRNAs are used as therapeutic targets for sepsis. For example, Wu et al. have depicted that lncRNA-HOTAIR facilitates the production of TNF-α in LPS-induced cardiomyocytes of mice with sepsis (Wu et al. 2016). Yong et al. have revealed that in sepsis, lncRNA-MALAT1 reduces BRCA1 expression by targeting EZH2, thereby accelerating skeletal muscle cell apoptosis and inflammation (Yong et al. 2020). KCNQ1 opposite strand/antisense transcript 1 (KCNQ1OT1) is located at 11p15.5 of KCNQ1 and is 91 kb in length (Kanduri 2011), and it has been proved to be closely related to the occurrence of many diseases. For example, lowering the level of KCNQ1OT1 alleviates myocardial I/R damage after acute myocardial infarction (Li et al. 2017). KCNQ1OT1 interacts with miR-140-5p to promote cholangiocarcinoma cell behavior, such as proliferation, migration, and invasion (Sun et al. 2018a). However, the biological function of KCNQ1OT1 in sepsis had not been fully explored, and its exact molecular mechanism needed to be further elucidated.

Herein, we expect to investigate the involvement of lncRNAs and miRNAs in sepsis. It was speculated that exosomal KCNQ1OT1 might play a vital role in the progression of sepsis by targeting the miR-154-3p/RNF19A axis.

## Materials and methods

### Animals and groups

A total of 18 male C57BL/6 mice, weighing 180–250 g, were purchased from the Laboratory Animal Centre of Nantong University (Nantong, China). And these mice were randomly divided into three groups: (I) control group (*n* = 6), treatment with saline; (II) cecal ligation puncture group (*n* = 6, CLP), underwent CLP surgery; (III) exosome group (*n* = 6, Exos), after CLP surgery, the mice were treated with exosome.

### Cecal ligation puncture (CLP)

The sepsis animal model was established by CLP surgery. Before surgery, the C57BL/6 mice were anesthetized with ketamine (50 mg/kg) and xylazine (5 mg/kg). After disinfection, a midline incision was made in the abdomen. The cecum was then sectioned externally, ligated distally, and punctured once with a size 21 needle. Finally, the mice were resuscitated with saline.

### Cell culture and treatments

BMMSCs were isolated from the C57BL/6 mice. In short, the mice were anesthetized and sacrificed, the skin was disinfected with 75% ethanol, the skin and muscles of both hindlimbs were cut rapidly under sterile conditions, the femur and tibia were removed, and the cells were isolated from the bone marrow of the femur and tibia; then, the extracted cells are then purified and identified. Human umbilical vein endothelial cells (HUVECs) were obtained from American Type Culture Collection and mouse aortic endothelial primary cells were obtained from CLP model mice. All cells are cultured in an environment with room temperature and saturated humidity.

### Isolation of exosomes

Exosomes were extracted from the supernatant of BMMSCs by ultracentrifugation. Briefly, the supernatant was filtered with SteritopTM 0.22-m sterile membrane, and then centrifuged at 100,000 × g for 1.5 h. Subsequently, the precipitate was then re-suspended at PBS (Aladdin) and centrifuged until the final volume was reduced to about 300 µL. Finally, the purified exosomes were stored at − 80 °C for later use.

### Observation of exosomes by transmission electron microscopy (TEM)

First, the purified exosomes were stained with 2% sodium phosphowolframate for 15 min, and then washed with PBS (Aladdin). Dried for 0.5 h at room temperature, exosomes were stained with 1% uranyl acid and observed using a TEM (Hitachi H7500 TEM, Tokyo).

### Nanoparticle tracking analysis (NTA)

The size distribution and concentration of the isolated exosomes were detected by NanoSight LM10 (NanoSight, UK). First, the exosome was dilute to 10,000 times with PBS (Aladdin) and mix upside down. Then, the diluted exosome was tested, photographed, and recorded under professional guidance.

### Western blot

The target cell protein was obtained by the RIPA method, and the protein concentration was determined by a BCA kit (Sigma). Then, 50 µg prepared protein was taken for gel electrophoresis separation, and the isolated protein was electrically transferred to the PVDF membrane (Roche, Switzerland). After being blocked with 5% nonfat dry milk, the PVDF membrane (Roche, Switzerland) was subjected to incubation with primary antibodies: CD63 (1:1000, ab134045, Abcam), CD9 (1:1000, ab92726, Abcam), HSP70 (1:1000, ab2787, Abcam), and VEGF (1:1000, ab32152, Abcam) at 4 °C overnight. On the following day, the membrane was incubated with the secondary antibody at 37 °C for 45 min. After washing the membrane film with TBST (Solarbio), the luminescent solution was added and exposed in the gel imaging system. The protein content was analyzed using Quantity-One software.

### Bioinformatics

StarBase (http://starbase.sysu.edu.cn/) was applied to speculate the target regulated by KCNQ10T1. miRWalk (http://www.umm.uni-heidelberg.de/apps/zmf/mirwalk/index.html), miRDB (http://www.mirdb.org/), and ENCORI (http://starbase.sysu.edu.cn/panCancer.php) were applied to predict the target genes regulated by KCNQ10T1 and miR-154-3p.

### Fluorescence labeling

Following the instructions of the PKH26 Red Fluorescent Cell Linker Mini Kit (Sigma), the purified exosomes were labeled under dark conditions. After 24 h of culture, PKH26 were co-cultured with HUVECs. Then, the labeled exosomes were detected by immunofluorescence assay.

### Cell Counting Kit-8

In brief, the cells adjusted to the appropriate concentration were inoculated on 96-well plates and treated accordingly. Next, each well was added with CCK-8 solution and incubated for 2 h in the dark. Finally, the absorption value at 450 nm was measured by a microplate reader (Rayto, RT6000).

### 5-Ethynyl-2-deoxyuridine (EdU) assay

The proliferation ability of the cells was detected using an EdU cell proliferation detection kit (RiboBio). Briefly, after different treatments, HUVECs were inoculated in 96-well plates for 48 h. The EdU solution was diluted with cell complete medium at the ratio of 1000:1 to prepare an appropriate amount of 50 µM EdU culture medium. Each well was added 300 µL EdU medium and then incubated for 2 h. After cell fixation, Apollo staining, and DNA staining, the proliferation activity of cells was observed and detected under a fluorescence microscope (Olympus).

### Wound healing assay

First, HUVECs were inoculated in a 6-well plate for 24 h. When the cells were fully fused, the pipette tip was applied to create a scratch wound on the confluent cells in the center. The migration and cell movement of the entire wound area were observed with an inverted optical microscope (Oberkochen, Germany), and the images were taken at 48 h with a camera connected to the microscope (SonyCyber shot, Shanghai Suoguang Visual Products Co., Ltd., China). The cell migration ability was statistically analyzed according to the cell healing.

### Transwell assay

HUVECs were firstly adjusted to the appropriate concentration. Subsequently, the upper chamber was added with adjusted cells, whereas the lower chamber was replaced by a medium supplement of 15% FBS (Avantor). After 24 h, 4% paraformaldehyde (Biorbyt) was used for fixation and 0.1% crystal violet (Aladdin) for staining, respectively. Five fields were randomly selected under an inverted microscope (Olympus) to represent the invasion ability of cells in each group.

### Measurement of interleukin (IL)-6 and IL-8 levels by ELISA

The concentrations of IL-6 and IL-8 in the HUVECs and CLP model mice were assessed using a commercially available ELISA kit (USCN Business Co., Ltd, China).

### Survival rate analysis

After mice underwent CLP surgery, the number of mice in each group was observed and counted for survival analysis. The death time and number of these animals were observed and recorded by a researcher who completely did not know the study.

### Hematoxylin–eosin (HE) staining

The aortic tissues were fixed with formaldehyde (10%) for 24 h, and then, tissues were placed in a 5% nitric acid decalcification solution for 3–5 days. After washing with water, routine dehydration, transparency, paraffin immersion, embedding, and sectioning, the tissue sections were stained with hematoxylin and eosin. Finally, the pathological changes in myocardium tissues were observed under microscope.

### Quantitative real-time PCR

TRIpure (Invitrogen, USA) was applied to isolate the sample RNA and PrimeScript RT kit (TaKaRa, Otsu, Japan) was used for reverse transcription. After the RNA was prepared, the expression level was detected with FAST SYBRTM Green Master Mix, and GAPDH was set as internal parameter. 2^−ΔΔCt^ methods represented the fold changes of gene expression and the experiments were conducted three times.

### Cell transfection

sh-NC, sh-KCNQ10T1, sh-RNF19A, NC mimic and miR-154-3p mimic, and miR-154-3p inhibitor were constructed by RiboBio corporation (Guangzhou, China). When the confluence rate of HUVECs reached 70 to 80%, the transfection was conducted by using Lipofectamine 2000 (Invitrogen, USA).

### Luciferase reporter assay

First, wt-KCNQ10T1, mut-KCNQ10T1, wt-RNF19A, and mut-RNF19A were conducted by QuikChange Site-Directed Mutagenesis Kit (Agilent Technologies). Then, HUVECs were incubated onto 24-well plates and co-transfected with wt-KCNQ10T1 or mut-KCNQ10T1 and wt-RNF19A or mut-RNF19A and miR-154-3p mimic or mimics-NC via Lipofectamine 2000. Finally, the luciferase activity was measured via the Dual-Glo Luciferase Assay System (Promega, USA).

### Statistical analysis

All the data were analyzed by SPSS 19.0. One-way ANOVA followed by Dunnett’s multiple comparisons was applied to assess the differences between the groups. Survival analysis was evaluated by the Kaplan–Meier method. *P* < 0.05 indicated a significant difference between groups.

## Results

### The characteristics of the exosomes extracted from BMMSCs

**Fig. 1:**
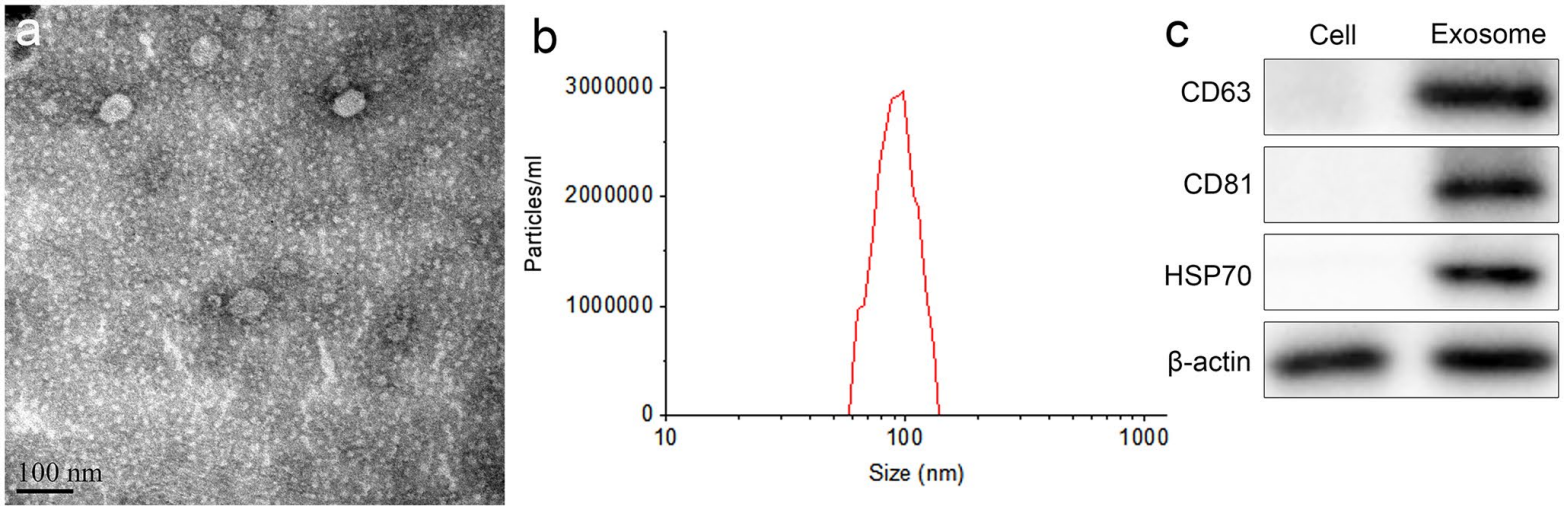
The characteristics of the exosomes extracted from BMMSCs. **a** TEM was applied to observe the morphology of exosomes extracted from BMMSCs; **b** NTA was used to analyze the size distribution of the extracted exosomes; **c** western blot was conducted to detect the surface antigens CD63, CD90, and HSP70 in exosomes

As shown in Fig. 1a, under TEM, exosomes derived from BMMSCs appeared round or oval. In Fig. 1b, NTA data showed that the average diameter of the exosome was 100 nm. Western blot assay revealed positive expression of CD63, CD90, and HSP70 in exosomes (Fig. 1c). These data indicated that the exosomes from BMMSCs were successfully extracted.

### Exosomes derived from BMMSCs inhibit LPS-induced proliferation, migration, and invasion of HUVECs

**Fig. 2:**
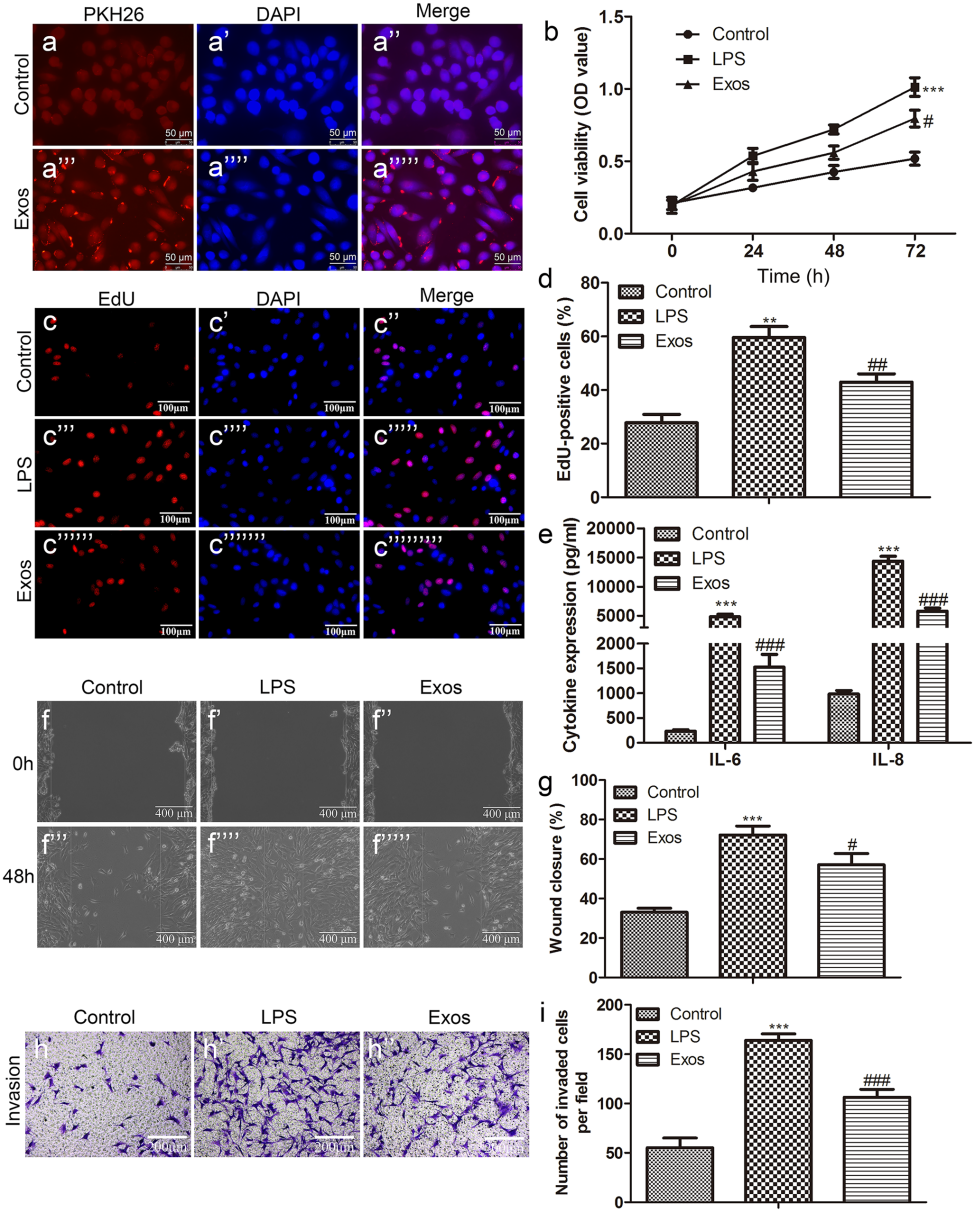
Exosomes derived from BMMSCs inhibit LPS-induced proliferation, migration, and invasion of HUVECs. **a** Fluorescence labeling was used to detect the internalization of exosomes in HUVECs (scale bar: 50 µm); the proliferation activity of HUVECs in the control, the LPS, and the Exos groups was observed by **b** CCK-8, **c** EdU, and **d** quantification (scale bar: 100 µm); **e** ELISA was conducted to test the levels of IL-6 and IL-8 in the cell supernatant. The migration and invasion of HUVECs in the control, the LPS, and the Exos groups were evaluated by **f** wound healing (scale bar: 400 µm) and **g** quantification and **h** Transwell and **i** quantification (scale bar: 200 µm)

To investigate the uptake and internalization of BMMSC-derived exosomes into recipient cells, HUVECs were treated with exosomes labeled with PKH26 red fluorescence. After 24 h, we observed that 90% of the exosomes were integrated into the cytoplasm, while in HUVECs labeled directly with PKH26, red fluorescence spread throughout the cell (Fig. 2a). Furthermore, we tested the biological effects of exosomes on HUVECs. Proliferation assays showed that LPS significantly induced the proliferation of HUVECs in comparison to control. In contrast, exosome treatment resulted in a remarkable reduction in HUVEC activity (Fig. 2b–d). ELISA results revealed that exosomes reduced the levels of IL-6 and IL-8 in the cell supernatant (Fig. 2e). Similarly, in the migration and invasion experiments, we found that the exosomes extracted from BMMSCs sharply slowed down the migration and invasion of cells induced by LPS (Fig. 2f–i).

### Exosomes derived from BMMSCs alleviate sepsis symptoms in CLP model mice

**Fig. 3:**
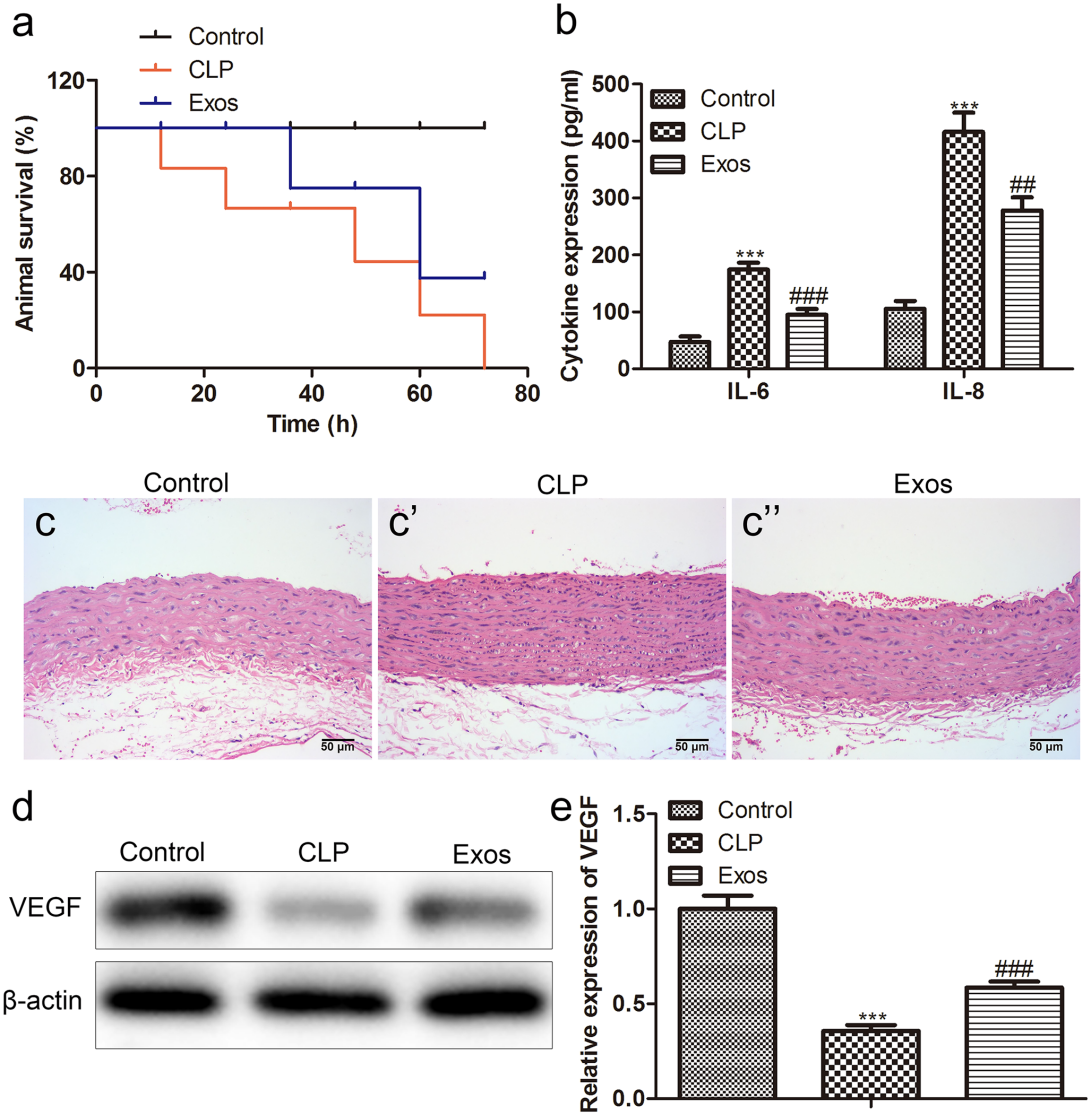
Exosomes derived from BMMSCs alleviate sepsis symptoms in CLP model mice. **a** Survival rate analysis of different groups of animals; **b** the levels of IL-6 and IL-8 in the cell supernatant were evaluated by ELISA; **c** the area of cross-sectional aortic necrosis in CLP mice was identified by HE staining (scale bar: 50 µm); **d** western blot; and **e** quantification was applied to quantitatively analyze the protein expression of VEGF in different groups

We demonstrated in vitro that exosomes inhibited LPS-induced sepsis. To further investigate the therapeutic effect of exosomes on sepsis, we conducted an in vivo experiment on CLP model mice. As shown in the survival analysis in Fig. 3a, CLP reduced the survival rate in mice. However, when these mice were treated with exosomes, the survival rate was significantly increased in model mice. In ELISA, decreased expression of IL-6 and IL-8 was found in the Exos group compared with the CLP group (Fig. 3b). Next, we extracted mouse aortic tissues to evaluate the effect of exosomes on the aortic tissue of CLP mice. HE staining showed that exosome treatment significantly reduced the necrotic area of the aortic cross-section of CLP mice (Fig. 3c). VEGF was an effective stimulator of endothelial cell permeability, and its elevated level was closely associated with sepsis. Therefore, we extracted aortic endothelial primary cells from CLP model mice to observe the changes in intracellular VEGF. As expected, the protein level of VEGF in the Exos group was evidently lower than group CLP (Fig. 3d, e).

### LncRNA-KCNQ1OT1 is involved in the inhibition of sepsis by BMMSC-derived exosomes

**Fig. 4:**
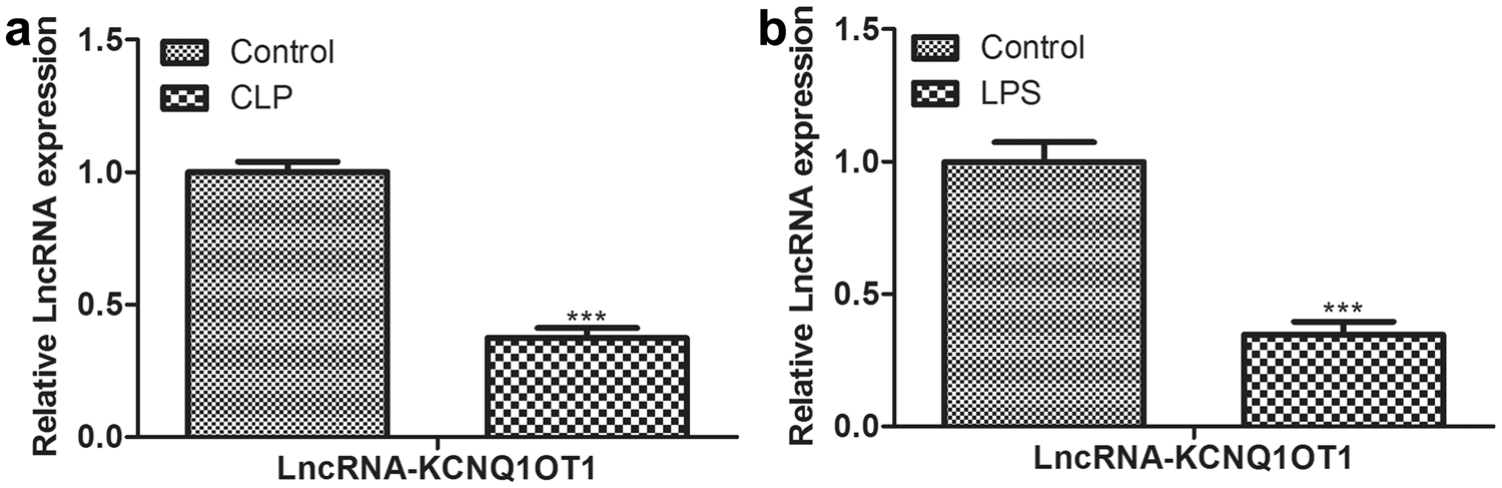
Expression levels of lncRNA-KCNQ1OT1. The expression of lncRNA-KCNQ1OT1 in **a** CLP model and **b** LPS-induced HUVECs was determined by qRT-PCR

**Fig. 5:**
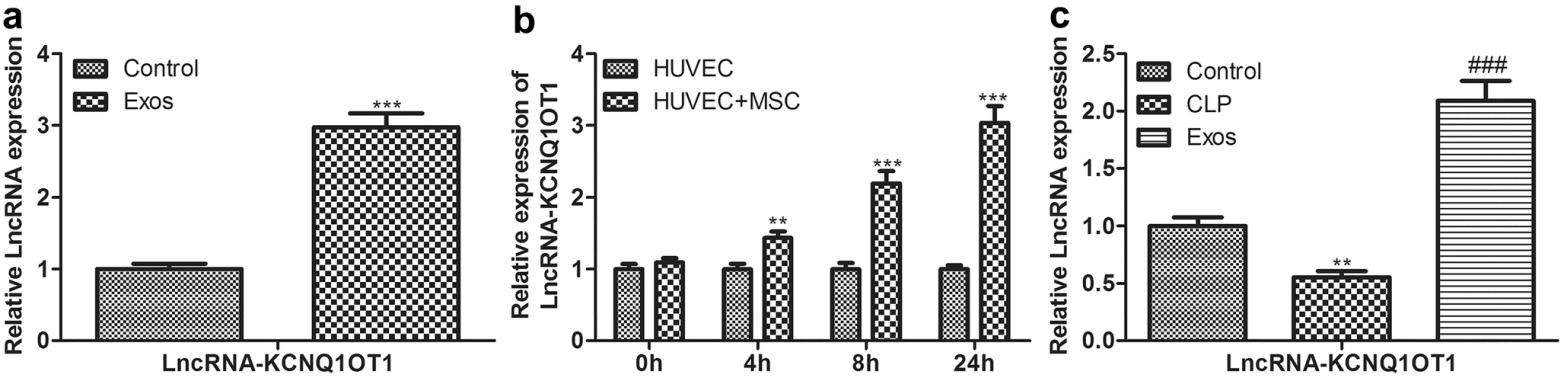
LncRNA-KCNQ1OT1 is involved in the inhibition of sepsis by BMMSC-derived exosomes. **a** The transcriptional activity of KCNQ1OT1 in the control group and Exos group was detected; **b** the transcriptional activity of KCNQ1OT1 in the HUVECs group and HUVECs + MSC group was detected; **c** the transcriptional activity of KCNQ1OT1 in the control group, CLP group, and Exos group was detected

As indicated in Fig. 4a and b, KCNQ1OT1 was lower in aortic endothelial primary cells from the CLP model and LPS-induced HUVECs than in the control group. On the contrary, the expression of KCNQ1OT1 was significantly up-regulated in exosomes, and the level of KCNQ1OT1 in HUVECs was significantly increased when BMMSCs and HUVECs were co-cultured (Fig. 5a and b). Furthermore, we compared the expression of KCNQ1OT1 in aortic endothelial primary cells from the CLP model before and after exosomal treatment and found that KCNQ1OT1 was evidently increased in exosomal treated cells (Fig. 5c).

### Overexpression of lncRNA-KCNQ1OT1 inhibits LPS-induced proliferation, migration, and invasion of HUVECs

**Fig. 6:**
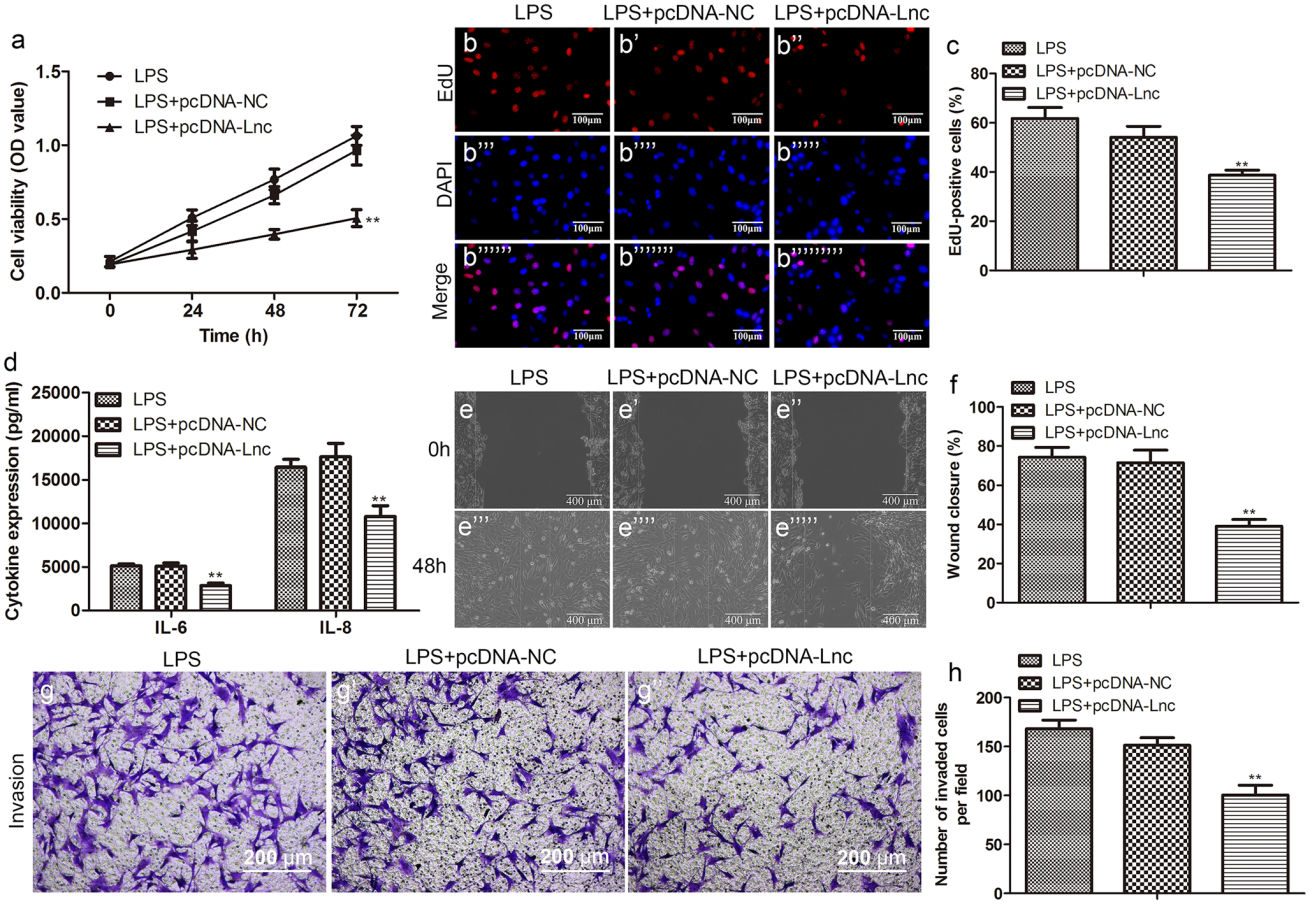
Overexpression of lncRNA-KCNQ1OT1 inhibits LPS-induced proliferation, migration, and invasion of HUVECs. After transfection with pc-KCNQ1OT1, the proliferation ability of LPS-induced HUVECs was measured by **a** CCK-8 and **b** EdU (scale bar: 100 µm) and **c** quantification; **d** the levels of IL-6 and IL-8 in the cell supernatant were determined by ELISA after transfection with pc-KCNQ1OT1; after transfection with pc-KCNQ1OT1, the migration ability and invasion ability of LPS-induced HUVECs were measured by **e** wound healing (scale bar: 400 µm) and **f** quantification and **g** Transwell (scale bar: 200 µm) and **h** quantification

To investigate the role of lncRNA-KCNQ1OT1 in sepsis, we overexpressed KCNQ1OT1. Next, the proliferation activity of HUVECs was detected by CCK8 and EdU assays, and the data showed that KCNQ1OT1 overexpression sharply inhibited LPS-induced cell activity (Fig. 6a–c). Meanwhile, we detected the levels of IL-6 and IL-8 in the HUVECs and observed that these inflammatory cytokines were sharply reduced in the LPS + pc-KCNQ1OT1 group in comparison to the LPS group (Fig. 6d). Furthermore, we examined the effect of pc-KCNQ1OT1 on the migration and invasion of HUVECs. As shown in the wound healing, the cell migration rate of group LPS + pc-KCNQ1OT1 was markedly lower than that of the LPS group (Fig. 6e and f). Similarly, the invasion efficiency of LPS-induced HUVEC cells was significantly inhibited after transfection with pc-KCNQ1OT1 (Fig. 6g and h).

### MiR-154-3p is the target of lncRNA-KCNQ1OT1

**Fig. 7:**
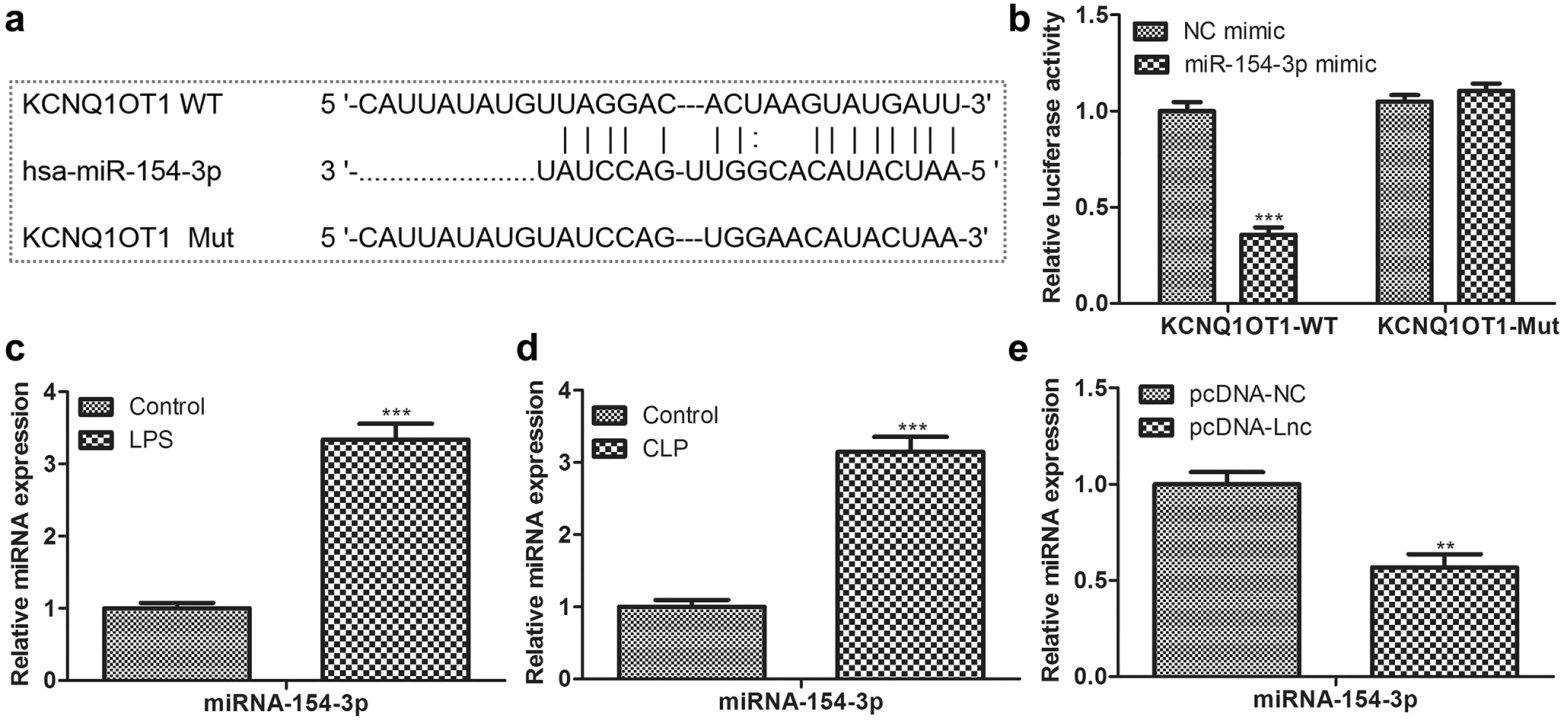
MiR-154-3p was the target of lncRNA-KCNQ1OT1. **a** The complementary binding region; **b** KCNQ1OT1-wt or KCNQ1OT1-mut were co-transfections with miR-154-3p; then, the luciferase activity was evaluated; the miR-154-3p expression in the **c** LPS-induced HUVECs and **d** mouse aortic endothelial primary cells from CLP model was detected; **e** MiR-154-3p mRNA expression in the pc-KCNQ1OT1 group was examined by RT-qPCR

To elucidate the molecular mechanism of lncRNA KCNQ1OT1 in regulating the progression of sepsis, we used starBase to predict its potential targets. As predicted, miR-154-3p was a putative target of KCNQ1OT1, and the binding sites are shown in Fig. 7a. To confirm this speculation, KCNQ1OT1-wt and KCNQ1OT1-mut were used to conduct luciferase reporter assay for verification. As depicted in Fig. 7b, the luciferase activity was significantly inhibited in the KCNQ1OT1-wt group, but there was no change in the KCNQ1OT1-mut group. Furthermore, we found that the mRNA level of miR-154-3p was sharply up-regulated both in LPS-induced HUVECs and in aortic primary cells extracted from the CLP model (Fig. 7c, d). Besides, the endogenous expression detection of miR-154-3p showed that pc-KCNQ1OT1 inhibited the transcription level of miR-154-3p, confirming that miR-154-3p bound with KCNQ1OT1 (Fig. 7e).

### Low expression of miR-154-3p inhibits proliferation, migration, and invasion of HUVECs induced by LPS

**Fig. 8:**
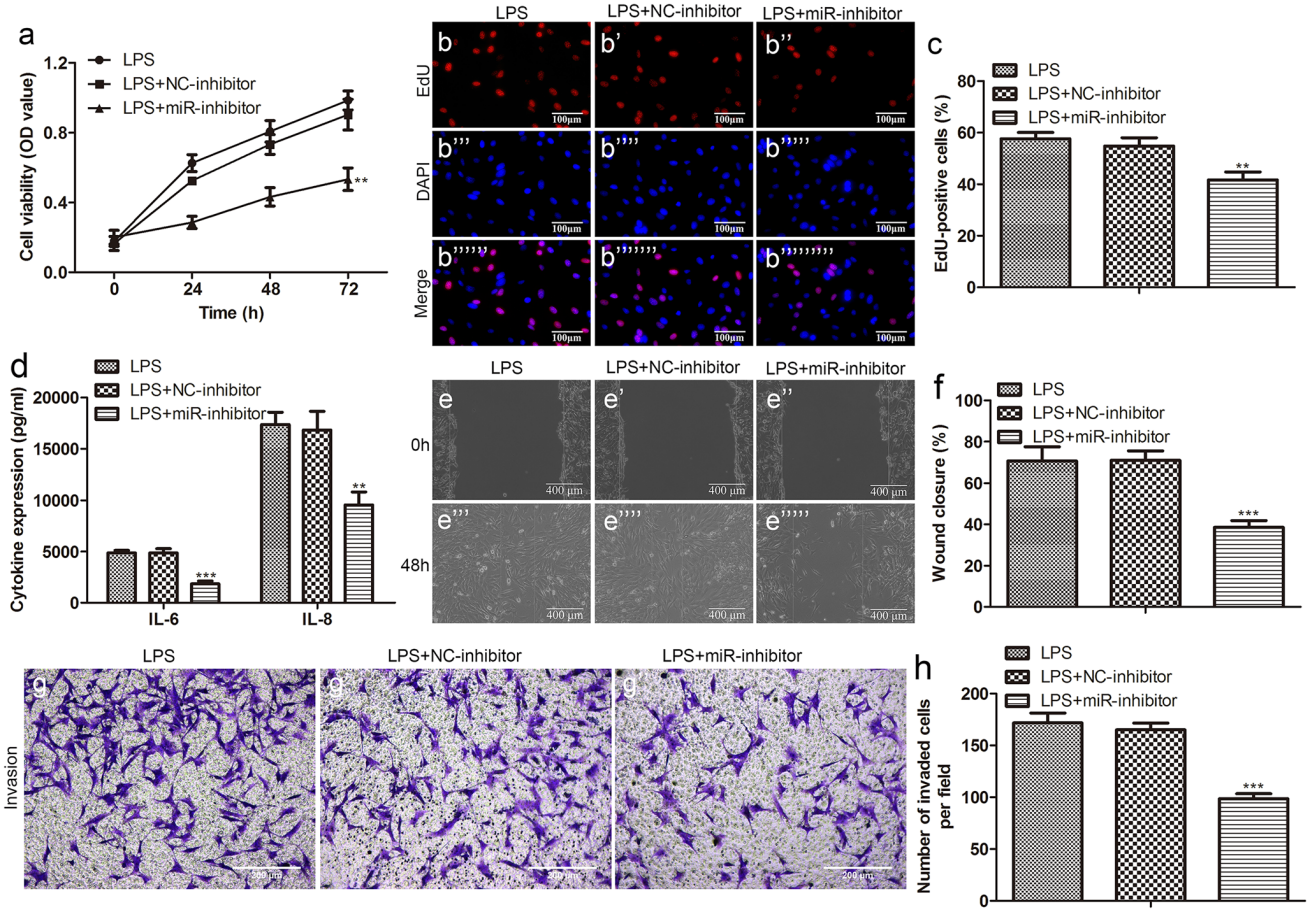
Low expression of miR-154-3p inhibited proliferation, migration, and invasion of HUVECs induced by LPS. After the addition of miR-154-3p inhibitor, the proliferation ability of LPS-induced HUVECs was measured by **a** CCK-8 and **b** EdU (scale bar: 100 µm) and **c** quantification; **d** the levels of IL-6 and IL-8 in the cell supernatant were determined by ELISA after the addition of miR-154-3p inhibitor; after the addition of miR-154-3p inhibitor, the migration ability and invasion ability of LPS-induced HUVECs were measured by **e** wound healing (scale bar: 400 µm) and **f** quantification and **g** Transwell (scale bar: 200 µm) and **h** quantification

To explore the role of miR-154-3p in sepsis, we constructed a low expression system of miR-154-3p by adding inhibitors and further detected its effects on cell biological functions. In CCK-8 and EdU assays, compared with LPS-treated HUVECs, the proliferation activity was sharply reduced after the addition of miR-154-3p inhibitor (Fig. 8a–c). In the ELISA assay, which measured inflammatory cytokine (IL-6 and IL-8) levels, the addition of miR-154-3p inhibitors remarkably decreased the levels of above inflammatory cytokines in the supernatant (Fig. 8d). In migration analysis, the relative wounding width of LPS + miR-154-3p inhibitor group was significantly greater than LPS group after 48-h culture in HUVECs (Fig. 8e, f). In Transwell assay, the number of invaded HUVECs per field in the LPS + miR-154-3p inhibitor group was significantly lower than that in LPS group (Fig. 8g, h).

### RNF19A is the target of miR-154-3p

**Fig. 9:**
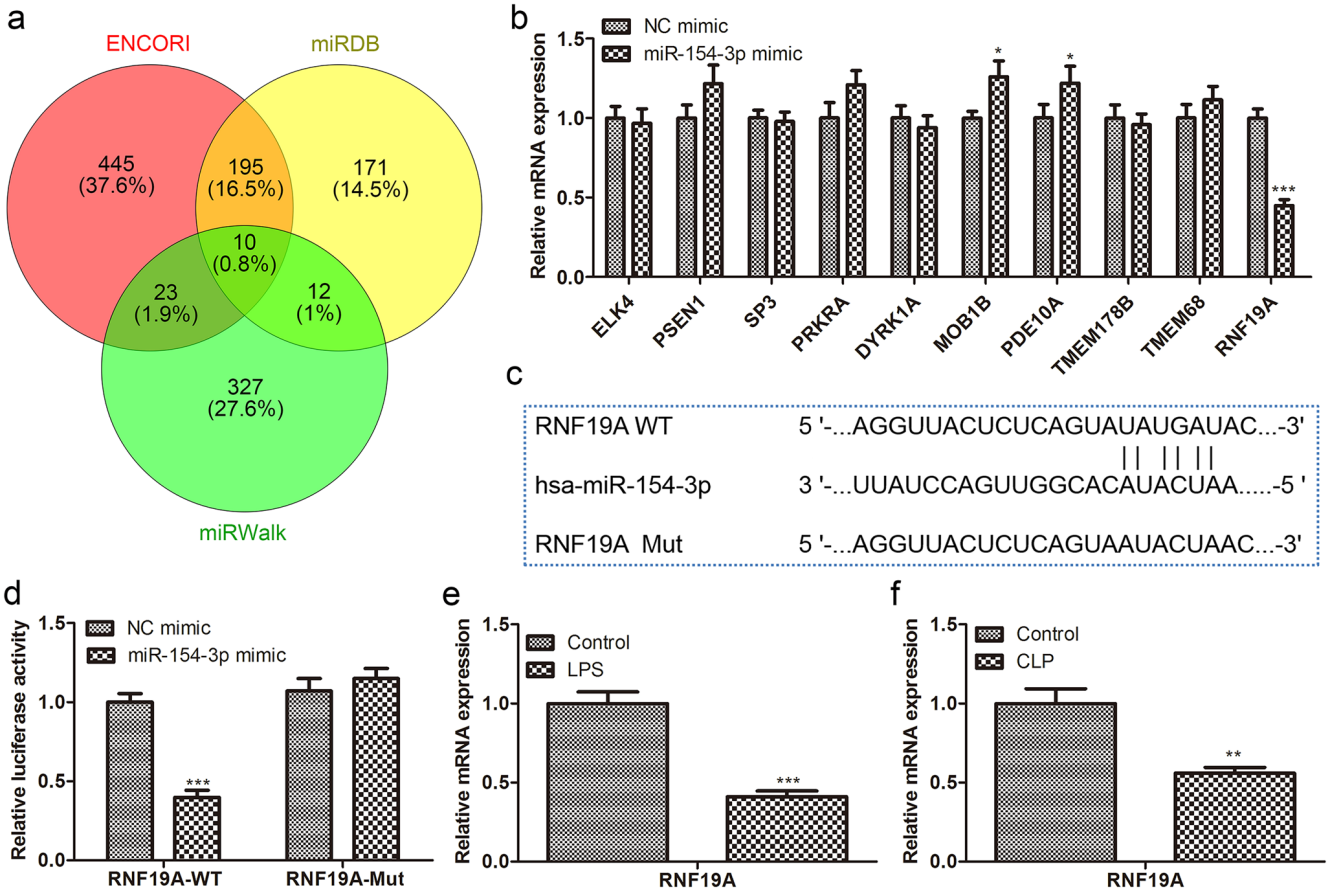
RNF19A was the target of miR-154-3p. **a** miRWalk, miRDB, and ENCORI were used to predict the target; **b** the mRNA levels of the screened target genes were quantitatively detected by RT-qPCR; **c** the binding site between miR-154-3p and RNF19A; **d** RNF19A-wt or RNF19A-mut were co-transfections with miR-154-3p mimic; then, the relative luciferase activity was evaluated; RNF19A mRNA expression in the **e** LPS-induced HUVECs and mouse aortic endothelial primary cells from **f** CLP model was examined by RT-qPCR

To clarify the regulatory mechanism of miR-154-3p in sepsis, we identified differentially expressed genes associated with sepsis via miRWalk, miRDB, and ENCORI algorithm, and a total of 10 overlapping genes were identified (Fig. 9a). Primers used for real-time PCR are shown in Table 1. Further quantitative analysis showed that only the RNF19A mRNA level was significantly reduced, suggesting that RNF19A might be the target gene of miR-154-3p involved in sepsis (Fig. 9b). The binding sites between miR-154-3p and RNF19A are presented in Fig. 9c. To verify the combination of the two, we carried out luciferase reporter assay and observed that the relative luciferase activity of RNF19A was significantly reduced after transfection with miR-154-3p mimic. However, when this binding site was mutated, overexpression of miR-154-3p did not affect luciferase activity (Fig. 9d). Besides, qRT-PCR results illustrated that transcription levels of RNF19A were remarkably reduced in the septic cell model (Fig. 9e, f).

**Table 1:** Primers used for real-time PCR

**Gene**	**Forward (5′-3′)**	**Reserve (3′-5′)**
KCNQ1OT1	CCCAGAAATCCACACCTCGG	TCCTCAGTGAGCAGATGGAGA
miR-154-3p	GTGGTACTTGAAGATAGGTT	TTGGTACTGAAAAATAGGTC
RNF19A	ACTGAACGGTTTAATCCT	CTGTTCCACAGCCTTCTC
ELK4	AACCAGCCTGCACGCGCT	TGCCCATCATTAGAGGTCCAACAG
PSEN1	TATGGCAGAAGGAGACCCG	TATGGCAGAAGGAGACCCG
SP3	CGCAGAAAGTCAGATGCCCT	TGGCTACCAGGCCTATGGAA
PRKRA	ACGAATACGGCATGAAGACC	TGGAAGGGTCAGGCATTAAG
DYRK1A	TCTGGGTATTCCACCTGCTC	GTCCTCCTGTTTCCACTCCA
MOB1B	TTCGGATGGCTGTCATGCTTCC	GCTGACATCACTGGACAACTCTC
PDE10A	GACCTCGCACTGTACTTTG	CTGGCCATAGTTTCGTCAC
TMEM68	GCGAATTCGCCATGATAGATAACAACCAAAC	CAGGATCCATGAGCCTTCTGCTCTTTAT
U6	CTCGCTTCGGCAGCACA	AACGCTTCACGAATTTGCGT
GADPH	GGTGAAGGTCGGTGTGAACG	CTCGCTCCTGGAAGATGGTG

*KCNQ1OT1 *KCNQ1 opposite strand/antisense transcript 1, *RNF19A *ring finger protein 19A, *PSEN1 *PTEN-induced putative kinase 1, *SP3 *specificity proteins 3, *PRKRA *interferon-inducible double-stranded RNA-dependent protein kinase 20 activator A, *MOB1B *Mps one binder kinase activator-like 1B, *PDE10A *phosphodiesterase 10A, *TMEM68 *transmembrane protein

### LncRNA-KCNQ1OT1 inhibits sepsis progression by targeting miR-154-3p/RNF19A

**Fig. 10:**
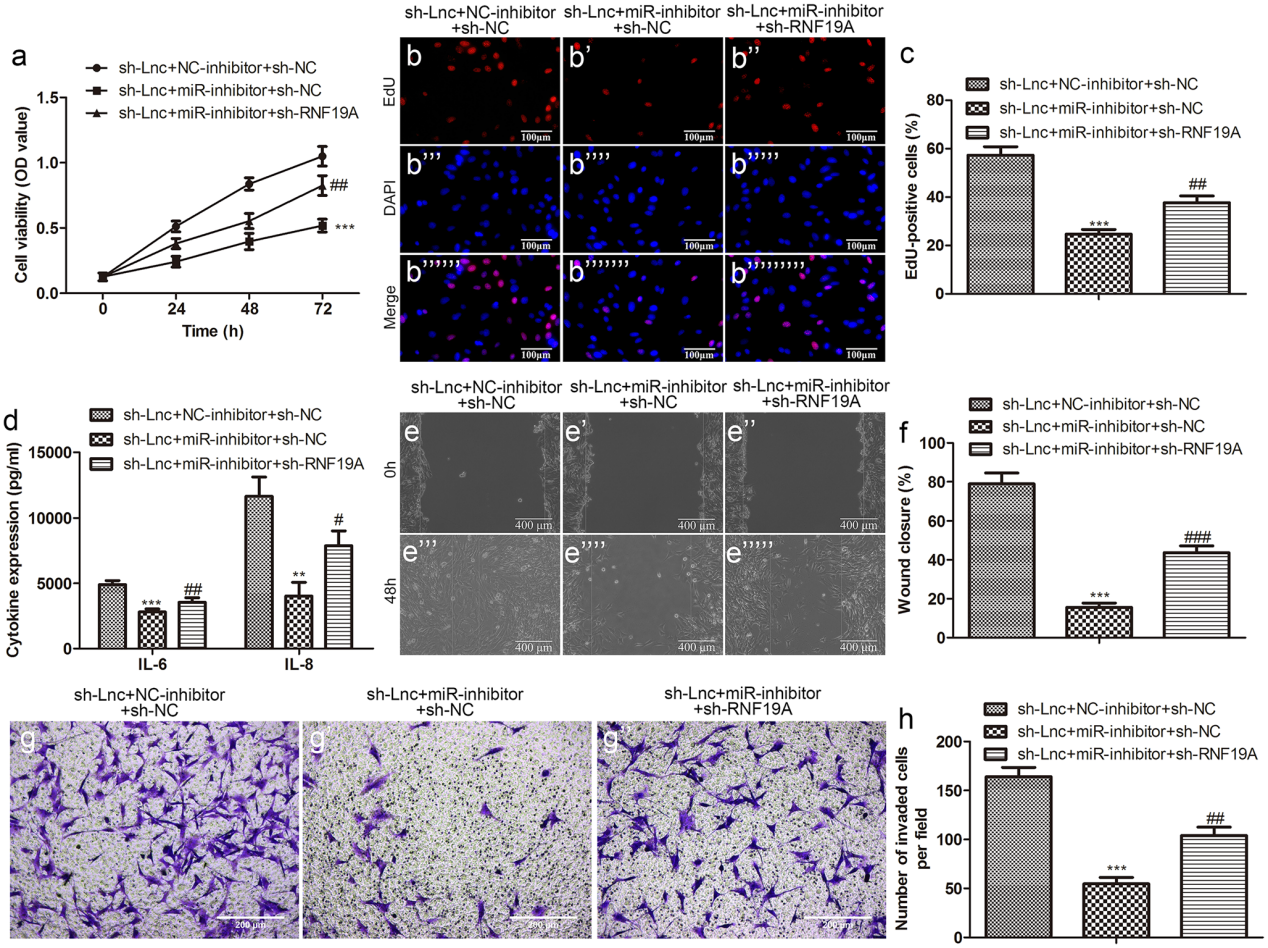
LncRNA-KCNQ1OT1 inhibits sepsis progression by targeting miR-154-3p/RNF19A. The proliferation ability of LPS-induced HUVECs in the indicated groups was measured by **a** CCK-8 and **b** EdU (scale bar: 100 µm) and **c** quantification; **d** the levels of IL-6 and IL-8 in the cell supernatant were determined by ELISA; the migration ability and invasion ability of LPS-induced HUVECs in indicated groups were measured by **e** wound healing (scale bar: 400 µm) and **f** quantification and **g** Transwell (scale bar: 200 µm) and **h** quantification

To further explore the interaction relationship between KCNQ1OT1, miR-154-3p, and RNF19A in the progression of sepsis, we transfected HUVEC cells with sh-KCNQ1OT1 + NC-inhibitor or sh-KCNQ1OT1 + miR-154-3p inhibitor or sh-KCNQ1OT1 + miR-154-3p inhibitor + sh-RNF19A. The proliferation activity of HUVEC cells co-transfected with sh-KCNQ1OT1 + miR-154-3p inhibitor was significantly lower than those transfected with pc-KCNQ1OT1 alone. However, when sh-RNF19A was added, the proliferation activity of HUVEC cells was sharply increased in comparison to the sh-KCNQ1OT1 + miR-154-3p inhibitor group (Fig. 10a–c). Similarly, the levels of IL-6 and IL-8 in group sh-KCNQ1OT1 + miR-154-3p inhibitor + sh-RNF19A were significantly higher than those in group sh-KCNQ1OT1 + miR-154-3p inhibitor (Fig. 10d). In cell metastasis assays (wound healing and Transwell), we still observed the same expression pattern, that is, transfection sh-KCNQ1OT1 + miR-154-3p inhibitor + sh-RNF19A increased the ability of cell migration and invasion compared with transfection sh-KCNQ1OT1 + miR-154-3p inhibitor (Fig. 10e–h).

## Discussion

When the polycystic body fuses with the plasma membrane into the extracellular space, exosomes from the endosomal membrane are released (Gyorgy et al. 2011). Exosomes are uniform in size, usually between 50 and 150 nm (Niu et al. 2017). In addition, exosomes can be used as therapeutics because they can express specific marker proteins (Andreu and Yanez-Mo 2014). Typically, these markers play a vital role in intracellular formation and transport as well as target cell recognition. In the current study, we extracted exosomes from BMMSC successfully. The exosomes were round or oval, with an average size of about 100 nm, and expressed positive surface marker antigens such as CD63, CD81, and HSP70. Recently, exosomes have received widespread attention due to their extensive participation in the progression of various diseases, such as inflammation, tumorigenesis, and angiogenesis (Ailawadi et al. 2015; Wang et al. 2014). In our research, we confirmed that BMMSC-derived exosomes significantly reduced sepsis toxicity and alleviated sepsis symptoms through in vivo and in vitro experiments. Sepsis is characterized by severe cytokine storms, in which large amounts of proinflammatory cytokines can be detected in the bloodstream (Essandoh et al. 2015). Consistently, our results showed that exosomes significantly reduced proinflammatory cytokines in the supernatant.

Recently, it has been reported that exosomes from BMMSCs affected cell proliferation and survival through the transport of lncRNAs (Yong et al. 2020). Moreover, lncRNAs have been proved to be novel gene regulators and prognostic markers for sepsis (Jiang et al. 2018). Therefore, exploring the mechanism of lncRNAs in sepsis was conducive to finding an effective therapeutic target. It was reported that KCNQ1OT1 was related to many diseases and abnormally expressed in various diseases. Jiang et al. have revealed that silencing KCNQ1OT1 expression regulates QT interval prolongation (Hu et al. 2018). Hu et al. have found that KCNQ1OT1 up-regulates the expression of ABCC1, thereby increasing the chemotherapy resistance of oxaliplatin to hepatocellular carcinoma (Sun et al. 2018b). In addition, KCNQ1OT1 is an important prognostic marker in diabetic retinopathy, osteolysis, and other diseases (Bartel 2004; Shao et al. 2019). However, the mechanism of KCNQ1OT1 in sepsis had not been fully elucidated. In the present study, we found that KCNQ1OT1 expression was low in the sepsis model, and overall survival was relatively low. On the contrary, it was highly expressed in exosomes. More notably, HUVEC and BMMSC co-transfection significantly up-regulated KCNQ1OT1 expression. Further functional studies showed that overexpression of KCNQ1OT1 inhibited LPS-induced cell proliferation, metastasis, and inflammatory response. Taken together, our data indicated that BMMSC-derived exosomal KCNQ10T1 inhibited the progression of sepsis.

MicroRNAs (miRNAs) are small non-protein coding RNA molecules (Cheng et al. 2015). Since they play an important role in the regulation of proliferation, survival, apoptosis, and other biological processes, the abnormal expression of miRNAs is associated with the occurrence and development of many diseases (Chen et al. 2018; Xu et al. 2016). Previously, data has revealed that miR-154-3p is used as a tumor suppressor and an effective target for the treatment of breast cancer (Li et al. 2013). Another study has also shown that miR-154-3p was significantly down-regulated in ductal carcinoma in situ (Ma and Kan 2021). Besides, miR-154-3p has been reported in papillary thyroid carcinoma, which promoted the deterioration of papillary thyroid carcinoma (Takeuchi et al. 2004). However, there were few studies on the role of miR-154-3p in sepsis, l et alone its deeper regulatory mechanism. In this study, miR-154-3p was sharply up-regulated in the sepsis model as a downstream target of KCNQ1OT1. Further functional studies showed that low expression of miR-154-3p inhibited LPS-induced cell proliferation, metastasis, and inflammatory response. Collectively, we confirmed that KCNQ1OT1 acted as a sponge for miR-154-3p in the regulation of sepsis progression.

RNF19A, also known as Dorfin, has ubiquitin ligase activity (Park et al. 2015). Besides, RNF19A has an affinity for ubiquitin-conjugating enzymes (Huang et al. 2006). It has been reported that RNF19A is expressed in many organs, such as the kidney, liver, intestine, and central nervous system (Sone et al. 2010). A previous study shows that RNF19A is related to familial amyotrophic lateral sclerosis and Parkinson’s disease and inhibits neuron phenotype and motor neuron death (Rivkin et al. 2009). Besides, RNF19a is involved in sperm cell head formation, head-to-tail coupling, and tail development (Ho et al. 2016). In our study, we confirmed that RNF19A was significantly down-regulated in the sepsis model and that RNF19A was the downstream target gene of miR-154-3p involved in sepsis. Moreover, the rescue experiment suggested that KCNQ10T1 inhibited sepsis progression by targeting the miR-154-3p/RNF19A axis.

However, there are still some limitations in this paper, although the study does a lot of studies at the in vitro level; however, we do not do enough validation tests in vivo, on whether exosomes derived from BMMSCs can affect the proliferation and migration of tissue cells in vivo in CLP model mice, which still need further study. In addition, to further confirm the roles of LncRNA-KCNQ1OT1, miR-154-3p, and RNF19A in sepsis, it might make the results more convincing to further reverse the study of the effects of high expression LncRNA-KCNQ1OT1/miR-154-3p/RNF19A on sepsis model in rescue experiments.

## Conclusions

In this study, we mainly explored the functionality of BMMSC-derived exosomal KCNQ10T1 and its potential interactions with the miR-154-3p/RNF19A axis in sepsis. We found that KCNQ10T1 was down-regulated in the sepsis model and might regulate the miR-154-3p/RNF19A axis to participate in the regulation of sepsis progression.

## Data Availability

All data generated or analyzed during this study are included in this published article. The article has a pre-print at the Research Square (Yuan et al. 2022).

## Abbreviations

BMMSCs: Bone marrow mesenchymal stem cells
CCK-8: Cell Counting Kit-8
CLP: Cecal ligation puncture
EdU: 5-Ethynyl-2-deoxyuridine
HE: Hematoxylin-eosin
HUVECs: Human umbilical vein endothelial cells
IL: Interleukin
KCNQ1OT1: KCNQ1 opposite strand/antisense transcript 1
lncRNAs: Long non-coding RNAs
LPS: Lipopolysaccharide
NTA: Nanoparticle tracking analysis
qRT-PCR: Quantitative real-time PCR
TEM: Transmission electron microscopy
